# Integrated in silico MS-based phosphoproteomics and network enrichment analysis of RASopathy proteins

**DOI:** 10.1186/s13023-021-01934-x

**Published:** 2021-07-06

**Authors:** Javier-Fernando Montero-Bullón, Óscar González-Velasco, María Isidoro-García, Jesus Lacal

**Affiliations:** 1 Metabolic Engineering Group, Department of Microbiology and Genetics, Faculty of Biology, University of Salamanca, 37007 Salamanca, Spain; 2 Bioinformatics and Functional Genomics Group, IBMCC Cancer Research Center, Campus Miguel de Unamuno, 37007 Salamanca, Spain; 3 Institute for Biomedical Research of Salamanca (IBSAL), 37007 Salamanca, Spain; 4 Network for Cooperative Research in Health–RETICS ARADyAL, 37007 Salamanca, Spain; 5 Department of Clinical Biochemistry, University Hospital of Salamanca, 37007 Salamanca, Spain; 6 Department of Medicine, University of Salamanca, 37007 Salamanca, Spain; 7 Molecular Genetics of Human Diseases Group, Department of Microbiology and Genetics, Faculty of Biology, University of Salamanca, 37007 Salamanca, Spain

**Keywords:** Phosphoproteomics, Interactomics, RASopathies, Neurofibromatosis, Noonan syndrome, Mass spectrometry, Rare diseases

## Abstract

**Background:**

RASopathies are a group of syndromes showing clinical overlap caused by mutations in genes affecting the RAS-MAPK pathway. Consequent disruption on cellular signaling leads and is driven by phosphoproteome remodeling. However, we still lack a comprehensive picture of the different key players and altered downstream effectors.

**Methods:**

An in silico interactome of RASopathy proteins was generated using pathway enrichment analysis/STRING tool, including identification of main hub proteins. We also integrated phosphoproteomic and immunoblotting studies using previous published information on RASopathy proteins and their neighbors in the context of RASopathy syndromes. Data from Phosphosite database (www.phosphosite.org) was collected in order to obtain the potential phosphosites subjected to regulation in the 27 causative RASopathy proteins. We compiled a dataset of dysregulated phosphosites in RASopathies, searched for commonalities between syndromes in harmonized data, and analyzed the role of phosphorylation in the syndromes by the identification of key players between the causative RASopathy proteins and the associated interactome.

**Results:**

In this study, we provide a curated data set of 27 causative RASopathy genes, identify up to 511 protein–protein associations using pathway enrichment analysis/STRING tool, and identify 12 nodes as main hub proteins. We found that a large group of proteins contain tyrosine residues and their biological processes include but are not limited to the nervous system. Harmonizing published RASopathy phosphoproteomic and immunoblotting studies we identified a total of 147 phosphosites with increased phosphorylation, whereas 47 have reduced phosphorylation. The PKB signaling pathway is the most represented among the dysregulated phosphoproteins within the RASopathy proteins and their neighbors, followed by phosphoproteins implicated in the regulation of cell proliferation and the MAPK pathway.

**Conclusions:**

This work illustrates the complex network underlying the RASopathies and the potential of phosphoproteomics for dissecting the molecular mechanisms in these syndromes. A combined study of associated genes, their interactome and phosphorylation events in RASopathies, elucidates key players and mechanisms to direct future research, diagnosis and therapeutic windows.

**Supplementary Information:**

The online version contains supplementary material available at 10.1186/s13023-021-01934-x.

## Background

**Fig. 1:**
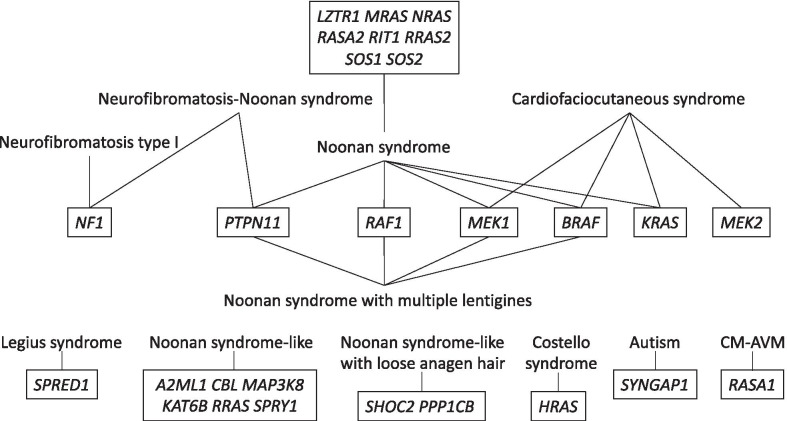
RASopathy genes. Neurofibromatosis type I, Legius and Costello syndromes, intellectual disability and people with autism spectrum disorder (Autism), and capillary malformation-arteriovenous malformation syndrome (CM-AVM) have just one protein as the cause of the disease, whereas the rest of RASopathies may be triggered by several defective proteins. In more than 90% of cases juvenile myelomonocytic leukemia (JMML) (not illustrated in the figure) is driven by alterations in *PTPN11*, *NRAS*, *CBL*, *KRAS* and *NF1* genes

RASopathies are a group of phenotypically overlapping syndromes caused by germline mutations that encode components of the RAS/MAPK signaling pathway, affecting growth and development [1, 2]. The RAS/MAPK signaling pathway is a chain of proteins in the cell that communicates a signal from a receptor on the surface of the cell to the DNA, and has major impact in human health [2–4]. These disorders include neurofibromatosis type 1 (NF1), Legius syndrome (LS), Noonan syndrome (NS), neurofibromatosis-Noonan syndrome (NFNS), Noonan syndrome-like (NSL), Noonan syndrome with multiple lentigines (NSML), formerly known as LEOPARD syndrome, Noonan syndrome-like with loose anagen hair (NSLSH) also known as Mazzanti syndrome, Costello syndrome (CS), cardiofaciocutaneous (CFC) syndrome, capillary malformation-arteriovenous malformation syndrome (CM-AVM), intellectual disability associated with autism spectrum disorder and juvenile myelomonocytic leukemia (JMML) [3–5]. To date, mutations in 27 genes have been proven as cause of these RASopathies (Fig. 1).

Neurofibromatosis type 1 (NF1) is caused by mutations in the *NF1* gene [6], whereas Legius syndrome, also known as NF1-like syndrome, is caused by mutations in the *SPRED1* gene [7]. Mutations in 13 genes so far, have been reported to underlie the Noonan syndrome (NS), the most genetically diverse RASopathy and the most common. Approximately 80% of individuals with NS harbor mutations in genes whose products are involved in the RAS/MAPK pathway including *PTPN11* [8–12] in about half of all cases, *SOS1* [8, 9, 11–14] in an additional 10 to 15%, *RAF1* [8, 9, 11, 12, 14–17] and *RIT1* [12, 18–20] in about an additional 5%. The remaining underlying genetic causes in nearly 20% of individuals with NS includes pathogenic variants in *BRAF* [8, 21], *KRAS* [8, 12, 14, 22, 23], *LZTR1* [12, 24–27], *MAP2K1* [23, 28, 29], *MRAS* [30–32], *NRAS* [12, 33–35], *RASA2* [29, 36], *RRAS2* [29, 37, 38] and *SOS2* [24]. Further clinical and genetic analysis is required to establish the pathogenic significance for some of these genes, including *RASA2*, SOS2 and *BRAF*. About 3% of all affected individuals with NS correspond to additional unidentified genes. Neurofibromatosis-Noonan syndrome (NFNS) is a rare condition with clinical features of both NF1 and NS. The major gene involved in NFNS is *NF1*, but co-occurring *NF1* and *PTPN11* mutations have been reported [29, 39].

Patients with NF1, and Noonan syndrome, have a higher risk of developing juvenile myelomonocytic myeloid leukemia (JMML) [40]. Recently considered as a bona fide RASopathy [4], JMML is a rare clonal myelodysplastic/myeloproliferative neoplasm of early childhood caused by hyperactive RAS signaling [41]. About 90% of patients with JMML harbor molecular alterations in 1 of 5 genes, all of them encoding RASopathy proteins. Genes that increase the risk of developing JMML include *NF1* (5–12%), *PTPN11* (37–38%), *KRAS* (17–18%), *NRAS* (14%) and *CBL* (9–18%) [42–44]. Minority genes responsible for JMML include *ARHGAP26* [45], *PCR2* and *RAC2* [46], *JAK3* and *SETBP1* [47, 48], and presumably *SPECC1* along with *CTSB* and *PDGFRB* [49].

Noonan syndrome-like is caused by a group of genes other than the common ones for NS, including mutations in *A2ML1* [50], *CBL* [9, 12, 51], *MAP3K8* [29], *MYST4/KAT6B* [52], *RRAS* [53] and *SPRY1* [8, 29]. Noonan syndrome with multiple lentigines (NSML) can be of 3 types, which are distinguished by their genetic cause. Type 1, corresponding to 90% of all cases, is caused by mutations in the *PTPN11* gene [8, 9, 11, 15]. Type 2 is caused by mutations in *RAF1* [15]. Type 3 is caused by mutations in *BRAF* [21]. Others are caused by mutations in *MEK1* [54], and in some cases the cause is unknown. Noonan syndrome-like with loose anagen hair (NSLSH) is caused by mutations in the *SHOC2* [8, 12, 14, 55, 56] and *PPP1CB* genes [57, 58].

Costello syndrome is caused by mutations in the *HRAS* gene [8, 11, 59]. The features of Costello syndrome overlap significantly with two of the RASopathies, NS and CFC. Likewise, CFC overlaps significantly with Costello and Noonan syndromes. CFC syndrome can be caused by mutations in several genes including *BRAF* [8, 9, 11, 14, 21, 23, 60] with approximately 75% of all cases, *MEK1* [8, 23, 61] and *MEK2* [9, 14, 23, 62, 63] with another 10 to 15%, and *KRAS* [23, 60, 64] with fewer than 5%.

SYNGAP1 was identified as a protein causing autosomal dominant intellectual disability type 5 [65], and as a causative RASopathy protein [26]. SYNGAP1-related intellectual disability and some rare mutations in *SYNGAP1* are associated with autism spectrum disorder [65, 66]. However, more basic research is needed to better understand the molecular and cellular functions of this protein [67].

On top of its major role in cancer [68], heterozygous-inactivating mutations in *RASA1* cause the autosomal dominant capillary malformation-arteriovenous malformation syndrome (CM-AVM) [69–71]. In this study we have included 5 more genes that do not belong to the classical RASopathy genes but were included in a gene panel towards the molecular diagnosis of Noonan syndrome and other RASopathies by Institut für Medizinische Genetik (https://www.medgen.uzh.ch/de.html): *ANKRD11* [72], *FGFR3* [73–75], *MEF2C* [76, 77], *SHOX* [78–81], and *SRCAP* [68, 82]. Also, their clinical features due to their molecular implications when mutated are common to most RASopathy syndromes.

Phosphoproteomics is a valuable approach to understand diseases linked to phosphorylation events, and it has a potential value for the clinics and for a personalized medicine [83, 84]. Classical immunoblot assays have been used to study phosphorylated amino acids in RASopathy proteins [85–87]. Whereas with the advent of more sensitive and accurate liquid chromatography–mass spectrometry (LC–MS) instrumentation, the number of large-scale mass spectrometry-based phosphoproteomic studies has swelled over the past decade [88], initiating its application to RASopathies [85, 89–92]. These techniques should allow to pinpoint biologically relevant phosphorylation events of utmost importance in the mechanisms of RASopathies [5]. However, only a few studies focused on their dysregulated phosphoproteins and their biological implications. Approaches mimicked mutations found in human samples associated to NS, NSML, and NF1 [85–87, 89–94]. In NF1 studies, *NF1*-null cells derived from malignant peripheral nerve sheath tumors (MPNST) showed RAS cascade hyperactivation typical of NF1. The interplay of H-Ras, N-Ras, and K-Ras produced a counter-inhibition of these classical Ras proteins, revealing an intense phosphoproteome remodeling [89]. Other studies were also conducted focusing on proteins such as CRMP-2 [93] and dynein IC2-C [85]. RAF1 [86], BRAF [94], PZR and SIRPa [87] are related to both NS and NSML, and changes in their phosphorylation were also observed. High-throughput screening strategies based on mass spectrometry have also been used to study NS and NSML [90–92], revealing dozens of dysregulated phosphoproteins.

The current state of knowledge about the RASopathy interactome is mainly based on an integrated network presented at genome, interactome, and phenome levels [1]; Twelve causative genes and clinical symptoms were collected from OMIM and NCBI GeneReviews databases for 6 syndromes: NS, NSML, NF1, CFC, Legius and Costello syndrome. In particular, they created an interactome network based on interactions between 12 proteins (PTPN11, SOS1, RAF1, BRAF, KRAS, NRAS, HRAS, MAP2K1, MAP2K2, SPRED1, NF1, and RIT1) of the RASopathies and another one based on their 10 first neighbors. However, we still lack a comprehensive view about the protein–protein associations involved in all RASopathy proteins and their neighbors. Mutations in some of these genes may drive RASopathies, cancer, or both in the same patient, but that is something that needs to be empirically tested. Phosphoproteomics data in RASopathies is still scarce and its potential to decipher crucial signaling pathways involved in this family of disorders needs to be considered. The application of LC–MS analysis, relying on high-sensitive nanoflow setups and phosphoprotein enrichment, is encouraged to unveil underlying molecular mechanisms affected in these syndromes, and suggest therapeutic targets for clinical implementation.

In this work, we aim to provide an up to date panel of proteins underlying RASopathies in order to identify protein–protein associations, and assign them to their cognate syndromes. The interactome was further analyzed in silico by binary interactions between the RASopathy proteins, a search for main hub proteins in the interactome and their classification based on GO terms for biological processes. We also carried out an analysis of phosphorylation level changes in the proteins from the RASopathy interactome integrating previous phosphoproteomic studies in the context of RASopathy syndromes. Finally, we analyzed the phosphosite abundance in causative RASopathy proteins in silico.

## Results

### RASopathy protein–protein associations

**Fig. 2:**
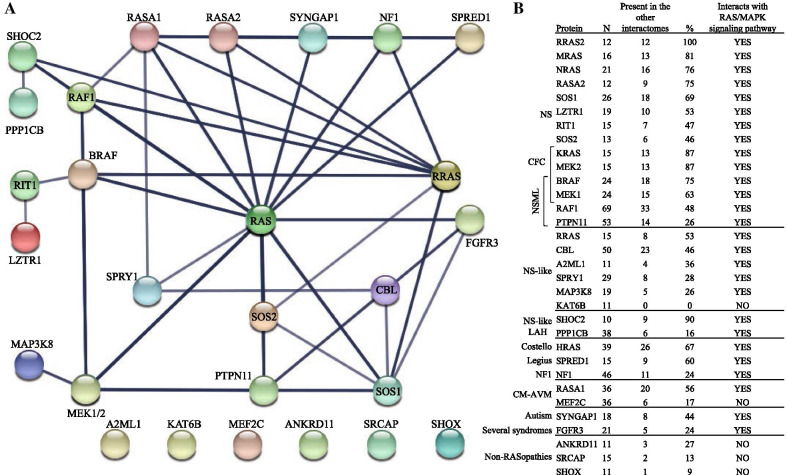
Association map of the 32 RASopathy proteins used in this study. Only 27 are shown since the RAS node includes HRAS, KRAS and NRAS, the RRAS node includes RRAS, RRAS2 and MRAS, and the MEK1/2 node includes MEK1 and MEK2. The thickness of the lines indicates the strength of data support according to STRING database. **A** Association map of the 32 RASopathy proteins used in this study. Corresponding proteins are shown in the figure. RAS includes six Ras proteins, whereas MEK1 and MEK2 are represented as MEK1/2. The thickness of the lines indicates the strength of data support according to STRING database. **B** Overlap of the interactome among the 32 RASopathy proteins. From left to right, protein names, number of direct partners, number of partners associated with other RASopathy proteins, and percentage of overlap. Last column indicates whether or not they interact with RAS/MAPK. Direct partners for each protein (N), were obtained from the interactome generated in this study including 27 RASopathy proteins and their neighbors (listed in Table S1)

In order to identify protein–protein associations within the RASopathy proteins we used STRING database to download a subgraph composed of the initially 32 RASopathy proteins selected in this study. Among the panel of 32 RASopathy proteins, our results show that 6 proteins belong to the RAS family, 15 are directly associated with RAS, and 11 proteins do not seem to be associated with RAS (Fig. 2A). RAS includes the classical RAS (H-RAS, K-RAS and N-RAS) and the three RRAS (RRAS, RRAS2 and RRAS3/MRAS). The 11 proteins that do not directly associate with RAS are ANKRD11, MEF2C, SHOX and SRCAP (aforementioned as non-classical RASopathy proteins), KAT6B, CBL, MAP3K8 and A2ML1 (which cause NSL), LZTR1 and RIT1 (responsible for NS), and PPP1CB (which causes NSLSH). The proteins that associate with all RAS include but are not limited to BRAF, RAF1, as well as several guanine nucleotide exchange factors (GEFs) and GTPase-activating proteins (GAPs) that regulate RAS activity (Fig. 2A).

We then generated an interactome for the 32 RASopathy proteins including their direct interacting partners. This interactome contains a total of 765 proteins. Some of them were found associated to more than one RASopathy protein and therefore they appeared duplicated. When eliminating the duplicated proteins, we ended up with 511 unique proteins based on unique UniProt IDs (Additional file 1). On average, the overlap of the interactome among the 32 RASopathy proteins is 56%. According to our results, RRAS2 interactome fully overlaps with other RASopathy protein’s interactome, followed by SHOC2 (90%), MEK2 (87%) and RAS (87–53%) among other proteins (Fig. 2B). Interestingly, FGFR3 did show direct interaction with RAS, was previously shown to exert and impact on RAS-MAPK signaling pathway [73–75] and had an interactome overlap of 24% with other RASopathy protein neighbors (Fig. 2B). On the other hand, ANKRD11, MEF2C, SRCAP, SHOX and KAT6B does not interact with RAS/MAPK and they show a low overlap (Fig. 2B). Therefore, they were not considered for further analyses in this study. However, A2ML1 was not excluded because according to our results A2ML1 interactome has a protein–protein association overlap with the rest of the RASopathy interactomes above 25%, which is even higher than the one obtained for other RASopathy proteins. Also, A2ML1 may interacts with the RAS-MAPK signaling pathway; A2ML1 is known to bind to lipoprotein receptor-related protein 1 activating of the Ras/MAPK pathway through its association with SHC domain proteins and CBL during recruitment to the plasma membrane [95, 96]. Considering the above data, from the initial 32 RASopathy proteins we continue studying 27 proteins, excluding ANKRD11, KAT6B, MEF2C, SHOX and SRCAP from further analyses.

### The interactome of RASopathy proteins by syndrome

**Fig. 3:**
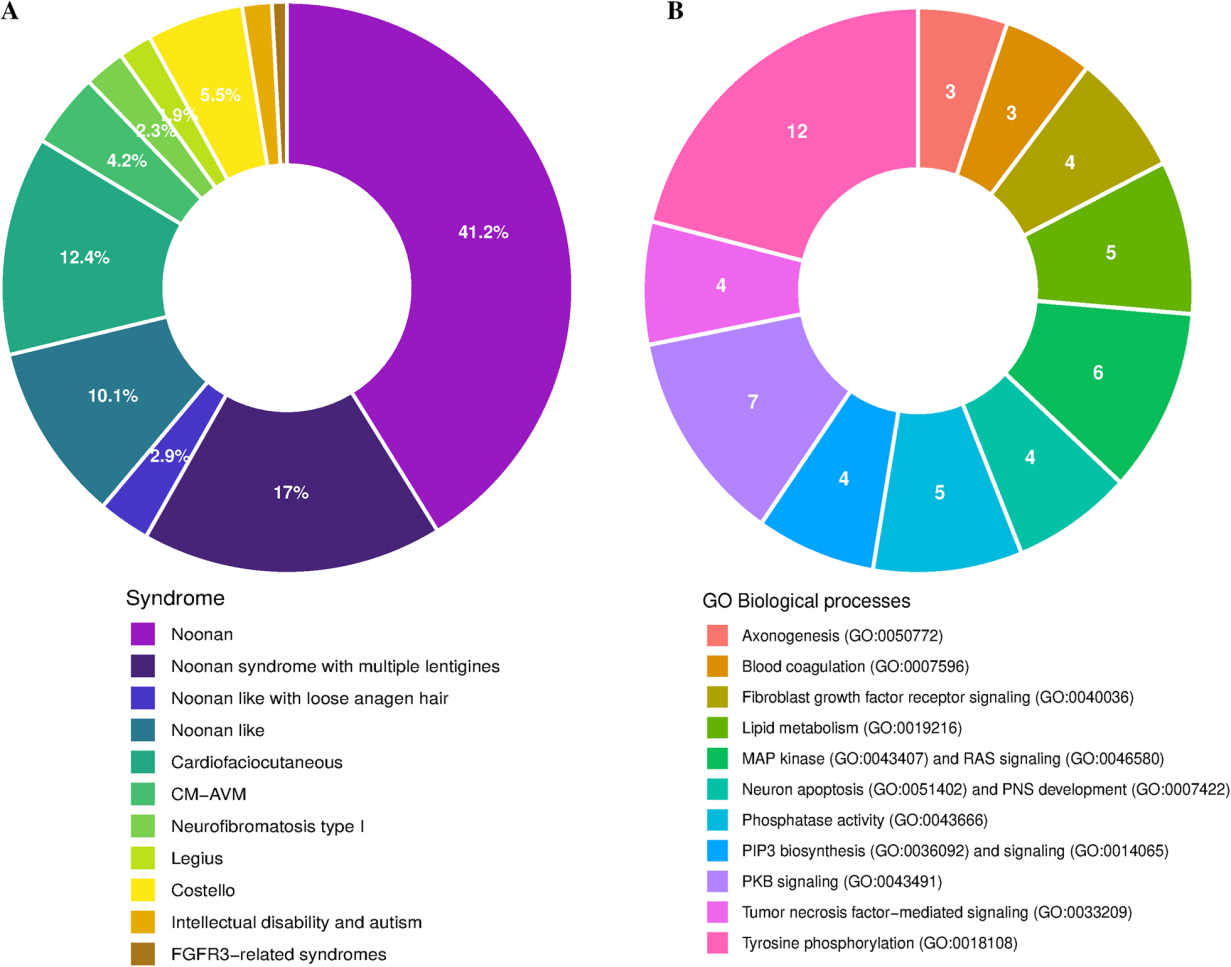
Analysis of the interactome and their implications in each syndrome and biological processes. **A** Comparison of the interactome of the different RASopathies. A total of 687 proteins were analyzed. The interactome of each RASopathy is intersected with the union of all the other RASopathies interactomes. **B** GO Biological processes overrepresentation test results using the 432 unique protein Ids of the interactomes on the PANTHER classification system

The interactome of 27 RASopathy proteins including their direct interacting partners, yield a total of 687 proteins (432 unique entries based on UniProt IDs). These proteins were assigned to their cognate syndromes in order to identify their occurrence by syndrome, purely reflecting the number of proteins associated with the syndrome (Fig. 3A). Our results show that the largest overlap correspond to NS with a total of 196 proteins (41.2%) shared with other RASopathy interactomes. On the other hand, FGFR3-related syndromes had the smallest overlap with only 4 proteins (0.8%) (Fig. 3A).

The analysis of all possible binary interactions between the interactome of each of the RASopathy proteins shows that 124 out of 351 binary interactions do not have in common a single protein (Additional file 2). Our results also show that 185 binary interactions share between 1 and 5 proteins, 38 between 6 and 10, and only 4 binary interactions share more than 10 proteins in their interactomes. This last set of binary interactions was found between HRAS and NRAS with 15 proteins, followed by BRAF and RAF1 with 14 proteins, HRAS and KRAS with 11 proteins, and HRAS and RRAS2 with 11 proteins (Additional file 2).

A search for main hub proteins in the interactome resulted in 12 nodes. The protein corresponding to the node with most associations with other genes within the RASopathy interactome is AKT1 with 199 associations reported. The second node with more interactions, 186 edges, corresponds to HRAS*.* PIK3R1 is the third with 124 known interactions. SRC yielded 118 interactions, SOS reported 109 interactions, GRB2 has 104, and CDC42 has 100 interactions. Other highly connected nodes found on the interactome network were NRAS with 94 interactions, SHC1 with 87, PPP2CA reporting 77, BDNF with 76 and JNK1 with 76 interactions.

An enrichment analysis based on GO terms for biological processes was done using the 432 unique proteins (Fig. 3B**)**. Interestingly the largest group of proteins correspond to proteins with phosphorylated tyrosine residues. Followed in abundance by PKB, RAS and MAPK signaling proteins, phosphatase activity and PIP3 biosynthesis and signaling proteins (Fig. 3B). We also found that several biological processes directly related with the nervous system may be altered, such as axonogenesis, peripheral nervous system development and neuron apoptosis.

### Analysis of dysregulated phosphoproteins in RASopathies

**Fig. 4:**
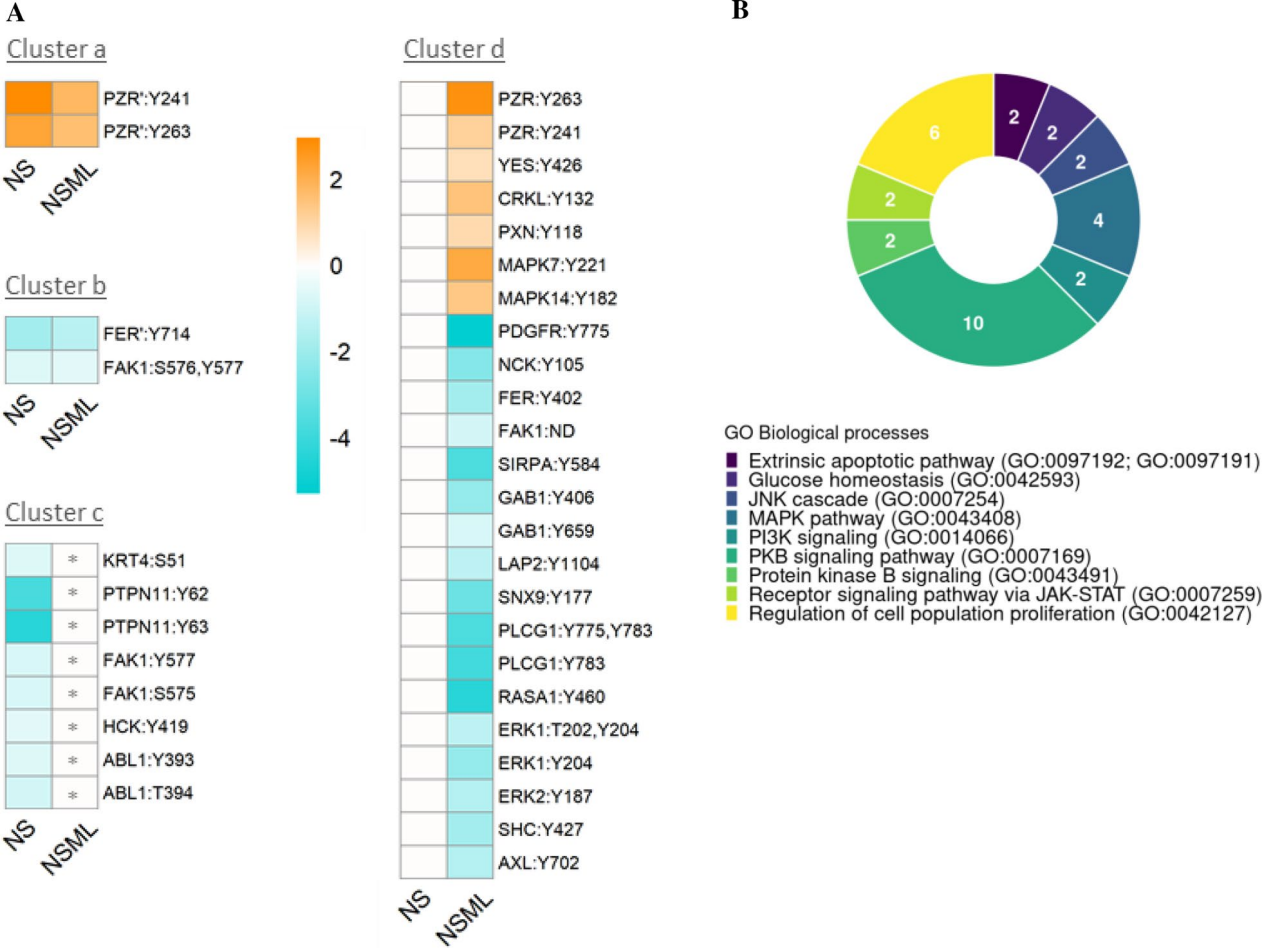
Analysis of dysregulated phosphosites in RASopathy interactome proteins in NS, NSML. **A** Heatmap representing the log2 (fold change) of phosphosites in NS and NSML versus control. Clustering analysis of the dysregulated phosphosites identified in phosphoproteomics and within the RASopathy interactome in either NS or NSML. An asterisk is added to zero values representing reported non-altered phosphorylation levels, to distinguish them from those representing just lack of data. In case of duplicated quantitative values from different studies, ‘ symbol was added [91]. **B** GO Biological processes overrepresentation test results using the 33 unique UniProt IDs from human dysregulated phosphosites reported in the RASopathy interactome on PANTHER

Functional annotation clustering of the interactome of the 27 RASopathy proteins and their interacting partners shows that 370 out of 432 proteins are annotated as phosphoproteins (Additional file 3). In order to study dysregulated phosphoproteins in RASopathies, protein phosphorylation level changes associated to RASopathies were compiled (Additional file 4) from nine phosphoproteomic (Fig. 4A) and twenty-seven immunoblotting studies (Table 1). The analysis of phosphorylation protein level changes within the RASopathy interactome comprises a total of 37 phosphoproteins (Additional file 5). In total and by syndrome, phosphoproteomic studies show that 8 phosphosites from 3 proteins are dysregulated in NF1, 53 phosphosites from 21 proteins in NS, 75 phosphosites from 31 proteins in NSML, 1 phosphosite in NSL, 2 phosphosites from 3 proteins in NSLH, 3 phosphosites from 2 proteins in LS, 4 phosphosites from 2 proteins in CS, 9 phosphosites from 3 proteins in CFC and 2 phosphosites from 2 proteins in JMML (crossing Additional file 1 and Additional file 4). Immunoblotting results show that dysregulation on several phosphoproteins account for more than one RASopathy, including ERK1/2 (Thr202/185 and Tyr204/187), AKT1 (Thr308 and Ser473), MEK1 (Thr292) and RAF1 (Ser259), which are the most co-occurring studied proteins along the syndromes (Table 1). For most RASopathies, phosphorylation of downstream effectors was upregulated compared to the control, including but not limited to AKT, MEK1 and ERK1/ERK2.

**Table 1:** Dysregulated downstream effectors in RASopathies based on immunoblotting

RASopathy	Up	Down
CFC	AKT1 ERK1 ERK2	AKT1 ERK1 ERK2 VEGFR2
CS	AKT1 ERK1 ERK2 P53 RAF1	nd
JMML	ERK1 ERK2 STAT5A	nd
LS	ERK1 ERK2	RAF1
NF1	ERK1 ERK2 Synapsin-1	DPYSL2
NS	AKT1 BRAF ERK1 ERK2 FAK1 FER GSK-3α IGF1R INSR JNK1 MEK1 MPZL1 P70S6K1 PECA1 RAF1 SHPS1 STAT5 VEGFR2	ANT1 GluN2B MEK1 RAF1
NSL	p90RSK3 S6K-alpha-1 S6K-alpha-2	nd
NSLSH	nd	ERK1 RAF1
NSML	AKT1 BRAF CAV2 EGFR ERK1 ERK2 FAK1 FER GSK-3α IGF1R INSR JNK1 MEK1 MPZL1 P70S6K1 PECA1 RAF1 SHPS1 STAT3 Tuberin	ERK1 ERK2 LKC MEK1 P70S6K1 RAF1 Vinculin

Reported phosphoproteins with dysregulated phosphorylation in mutated RASopathy causative genes samples compared to their wild-type counterparts. The disorders include cardiofaciocutaneous (CFC), Costello syndrome (CS), juvenile myelomonocytic leukemia (JMML), Legius syndrome (LS), neurofibromatosis type 1 (NF1), Noonan syndrome (NS), Noonan syndrome-like (NSL), Noonan syndrome-like with loose anagen hair (NSLSH) and Noonan syndrome with multiple lentigines (NSML). More information about the altered phosphosite, model organism and corresponding reference can be found in Additional file 4. Nd stands for not determined.

The most studied syndromes are NS and NSML, thanks to the availability of phosphoproteomics data. In NS the average fold change in upregulation is 2.61 and in downregulation 0.29, the highest fold change corresponds to PZR:Y241 (7.72) and the lowest to PTPN11:Y63 (0.02) with nearly a complete depletion of phosphorylation. In NSML the average fold change for upregulation is 2.29 and for downregulation 0.80, the highest value also corresponds to PZR:Y263 (6.7) whereas the lowest corresponds to PDGFR (0.02). These values demonstrate quantitively a strong dysregulation in phosphorylation. Figure 4 shows a comparison of quantitative values available for NS and NSML, that fulfil a threshold of 1.5 in both downregulation and upregulation events. Clustering analysis of the dysregulated phosphosites identified in phosphoproteomics and within the RASopathy interactome in either NS or NSML (Fig. 4A), results in a first cluster of 2 phosphosites (cluster a) that are upregulated both in NS and NSML. A second cluster of 2 phosphosites downregulated in both NS and NSML. Interestingly, there is a number of phosphosites found dysregulated in one syndrome but not in the other. Cluster c comprises phosphosites that are dysregulated in NS but not in NSML, while cluster d shows phosphosites dysregulated in NSML and not in NS. From the phosphoproteomic analysis done in NS and NSML, a total of 4 proteins in NS (CRKL, PZR, MAPK7 and MAPK14) and 1 in NSML (PZR) have a fold change greater than 2. On the other hand, 14 proteins in NS (AXL, LAP2, ERK1, ERK2, FER, GAB1, NCK, PDGFR, PLCG1, RASA1, SHC, SIRPA, SNX9 and PTPN11) and 1 in NSML (FER) have a fold change smaller than 0.5 in disease versus control. In Additional file 5, more details on the 37 dysregulated phosphoproteins within the interactome are annotated from UniProt, including gene and protein name, UniProt ID and protein function. Interestingly, dysregulation of four RASopathy proteins including BRAF, MEK1, PTPN11 and RAF1 happened to be similar in both NS and NSML. The two phosphosites in BRAF are upregulated in NS and NSML. RAF1 contains three upregulated phosphosites and one downregulated in both syndromes. PTPN11 contains a downregulated phosphosite in both NS and NSML, whereas two other residues are differentially phosphorylated. Residue Y62 in PTPN11 is downregulated in NS and upregulated in NSML, whereas Y63 is downregulated in NS but it was not altered in NSML. MEK1 is upregulated in phosphosite T292 in NS, while in NSML the regulation varies depending on the causative mutation.

### Analysis of phosphosite occurrence in RASopathy proteins

**Fig. 5:**
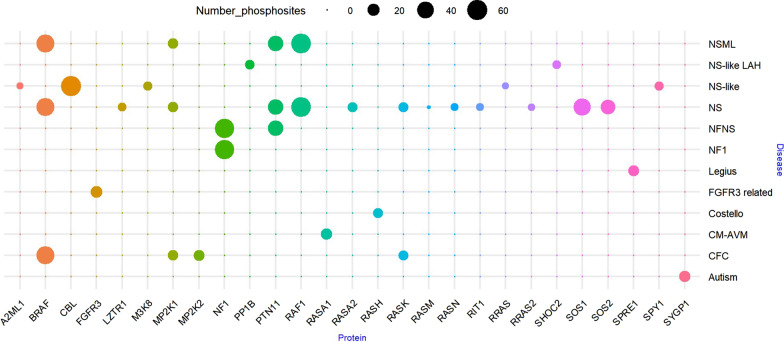
Identified phosphosites in RASopathy proteins and their cognate syndromes. The size of the bubbles represents the number of phosphosites for the corresponding protein, in the case of association to a cognate syndrome. Phosphosites for CBL, RASK, RASN, A2ML1 and PTN11 also overlap in JMML

Studies of the phosphoproteome in RASopathies are scarce, making difficult to evaluate how phosphorylation affects proteins. In order to have an overview of phosphorylation sites in RASopathy proteins, we used Phosphosite database to consider all documented phosphosites rather than only those with studied dysregulation. We analyzed the phosphosites for the 27 RASopathy proteins and their cognate syndromes. Information relies on low-throughput analysis (a total of 1041, 5.6%) and high-throughput analysis (17,656, 94.4%). Using this information, our results show that all RASopathies are associated to at least one phosphoprotein (Fig. 5). NS, with 13 proteins, contains the highest number of RASopathy proteins susceptible of phosphorylation, followed by NFNS (5 proteins) and JMML (5 proteins). Interestingly, all RASopathy proteins have residues potentially subjected to regulation by phosphorylation. BRAF, CBL, NF1, PTPN11, RAF1, SOS1 and SOS2 contain at least 20 phosphosites each, and they account for 326 of the phosphosites identified in RASopathy proteins (61% of the total). The rest of phosphosites (210) are distributed between the other 20 RASopathy proteins.

## Discussion

In this study we provide a global comprehensive picture of phosphoproteome remodeling in RASopathies, including but not limited to the most up-to-date information regarding the genes that cause the RASopathies. 5 (ANKRD11, MEF2C, SRCAP, SHOX and KAT6B) out of the initial 32 proteins were excluded from further analysis based on two factors: no direct association with RAS/MAPK signaling pathway and having an interactome with a protein–protein association overlap with the rest of the RASopathy´s interactomes below 16%. A2ML1 was not excluded because according to our results A2ML1 interactome has a protein–protein association overlap with the rest of the RASopathy´s interactomes above 25%, which is even higher than the one obtained for other RASopathy proteins. FGFR3 directly interacts with RAS, and our results suggest that 24% of its interactome is shared with other RASopathy proteins. This 24% is higher than the one observed for other RASopathy proteins, including but not limited to PPP1CB, and similar to NF1. FGFR3 was considered responsible for mosaic RASopathies [73], but has not been included in any RASopathy protein panel yet in clinical studies. FGFR3 has been found to influence the RAS/MAPK signaling pathway in many studies. FGFR3 mutations are frequent in superficial urothelial cell carcinoma (UCC) as oncogenic activation of FGFR3 is predicted to result in stimulation of the MAPK pathway [97]. MIP-1α (CCL3) is a downstream target of FGFR3 and RAS-MAPK signaling in multiple myeloma [98]. In hematopoietic cells, FGFR3 activates RSK2 to mediate activation of the MEK/ERK pathway [99]. Mutations in FGFR3 and PIK3CA, singly or combined with RAS and AKT1, are associated with AKT but not with MAPK pathway activation in urothelial bladder cancer [100]. However, in another study it was found that enhanced activation of FGFR3 is linked to Ras and MAPK activation; in particular the authors described a novel FGFR3/Ras mediated mechanism for acquired-resistance to B-RAF inhibition [101]. RAS and FGFR3 mutations in urothelial carcinoma are mutually exclusive and non-overlapping events which reflect activation of oncogenic pathways through different elements. Also, papillary cancers typically exhibit activation of the MAPK pathway, as a consequence of oncogenic mutations in *FGFR3* or *RAS* genes [102, 103]. Forced expression of FGFR3 mutants in NIH-3T3 cells resulted in cellular transformation and mitogen-activated protein kinase (MAPK) activation, resembling the transfection effects observed with activated HRAS [104, 105]. Activated FGFR3 seemed to be linked to RAS through adaptor proteins (that is, growth factor receptor-bound protein 2 (GRB2)- son of seven less (SOS) complexes) that are common to the RTK activation pathway [102]. These strong evidences of FGFR3 implications in the RAS/MAPK signaling pathway along with our in silico results of the interactome, let us suggest that FGFR3 disorders (thanatophoric dysplasia, achondroplasia, and hypochondroplasia) may be included among the RASopathies. On the other hand, KAT6B has no direct RAS interaction, poor direct association with RAS/MAPK signaling pathway with just one study so far [52] and its interactome is not associated at all with any other RASopathy protein. A translocation breakpoint 10q22.3 in a clinically diagnosed NS individual identified disruption of the *KAT6B* gene and hyperactivated MAPK signaling in humans and mice [52]. In particular, they found that functional studies using a patient-derived lymphoblastoid cell line causing *KAT6B* haploinsufficiency demonstrated an increase of RAS/MAPK pathway activity. Therefore, the authors postulated that altered expression of multiple genes associated with RAS/MAPK pathway regulation may be responsible for the increase in pathway activation and the NS-like phenotype [52]. However, that correlation has not been confirmed with more research so far. In a more recent study of a de novo heterozygous variant within exon 16 of *KAT6B* that was detected in a 7-months-old Chinese female infant, the patient presented symptoms of short stature, global developmental delay, and clinical features consistent with blepharophimosis mental retardation syndromes (SBBYSS, also called Ohdo syndrome) [106]. From a clinical point of view, the KAT6B phenotypic spectrum is broad and naming of the KAT6B-related disorders has been problematic, and suggested that considering this whole group as ‘KAT6B spectrum disorders' may be more helpful [107]. As expected, RAS proteins are the ones with the highest number of associations, although RASopathies related to SPRED1, MEK1/2, PTPN11, FGFR3 and SPRY1 may regulate RAS signaling differently, affecting primarily the classical RAS versus RRAS signaling.

We believe that further analysis is needed to determine the biological roles of the master nodes that we have identified in the RASopathy interactome. The functional characterization of these 12 nodes (AKT1, HRAS, PIK3R1, SRC, SOS, GRB2, CDC42, NRAS, SHC1, PPP2CA, BDNF and JNK1) may uncover implications and common patterns on the RASopathies signaling pathways, and their suitability as druggable targets. AKT is involved in many processes, including metabolism, proliferation, cell survival, growth and angiogenesis [108–111]**.** HRAS mutations are known to cause Costello syndrome [8, 11, 59]. Phosphatidylinositol 3-kinase plays an important role in the metabolic actions of insulin, and a mutation in this gene has been associated with insulin resistance [112]. *SRC* proto-oncogene may play a role in the regulation of embryonic development and cell growth [113]**.** GRB2 provides a critical link between cell surface growth factor receptors and the Ras signaling pathway [114, 115]. Cell division cycle 42 (CDC42) regulates signaling pathways that control diverse cellular functions including cell morphology, migration, endocytosis and cell cycle progression [116–118]. Interestingly, some of these nodes, such as SHC1:(SHC1:Y427), AKT1 (S473, T308) and JNK1 (T183, Y185) are also subjected to dysregulated phosphorylation according to our phosphoproteomic analysis.

Many of the signaling cascades associated to the RAS/MAPK pathway imply phosphorylation and dephosphorylation of proteins [5], and our results suggest that phosphoproteome remodeling in RASopathies is strongly relevant. The study of phosphosites is based on residues present in human and non-human model organisms that are extrapolated to the human sequences, so that dysregulated phosphosites may vary in human models and must be confirmed. A high proportion of the interactome (around 72%) are phosphoproteins, when compared to a 10% accepted value of phosphoproteins in the cytoplasm [89]. Further, all RASopathy proteins contain annotated phosphosites. Also, the gene ontology analysis of the RASopathy interactome and their dysregulated phosphoproteins shows mainly phosphorylation-related processes highlighting the PKB and MAPK pathways. Previous phosphoproteomics studies in RASopathies have revealed dysregulated phosphoproteins. Our results highlight several phosphoproteins dysregulated in NS, NSML and NF1 syndromes. The impact of a particular NS or NSML associated variant on downstream targets will depend first on whether the mutated RASopathy gene is linked particularly to NS (*LZTR1*, *MRAS*, *NRAS*, *RASA2*, *RIT1*, *RRAS2*, *SOS1*, *SOS2*) or both NS and NSML (*BRAF*, *MEK1*, *PTPN11*, *RAF1*). Second, mutations within the same causative gene linked to both NS and NSML may or may not have a different impact on downstream targets. Phosphoproteomic studies suggest that a higher proportion of phosphosites is upregulated (147 versus 47 downregulated), whereas immunoblotting suggest that most studied downstream effectors are upregulated, including but not limited to AKT1, BRAF, ERK, FAK, JNK, MEK and RAF. However, in some studies MEK and RAF have been also found downregulated in both syndromes. The relative variance in symptoms between RASopathies caused by activating signaling components in NS and NSML may suggest that there are different mechanisms at play. Systematic investigation of mutation strength across other pathway components rather than RAS, ERK or AKT may yield further insights into disease etiology. Subsequent studies on candidates highlighted by phosphoproteomics, as PZR protein in NS and NSML, have been already proved useful to explain crucial pathophysiological events, such as hypertrophic cardiomyopathy and cardiac fibrosis in NSML [119]*.* Phosphorylation of the RASopathy proteins RAF1 [86], PTPN11 [91, 92], and BRAF [94] is altered in NS and NSML due to mutations in their corresponding genes, whereas RASA1 altered phosphorylation is associated to mutant SHP2 models [92] and not to its own mutation. The most upregulated phosphoproteins from the RASopathy interactome in NS and NSML, except for CRKL, have already been proposed as key players in these syndromes, namely PZR [90] and Fer kinase [91]. Similarly, downregulated phosphorylation levels of DPLY2 in NF1 have been well studied in neuronal differentiation [93]. Remarkably, contradictory values for identical phosphosites between different studies is an intriguing reason to further evaluate.

Phosphorylation events in the RASopathy interactome remain largely unknown. Dysregulated phosphoproteins studies only reported data for NF1 [85, 89], NS [86, 90, 91, 94] and NSML [90, 91]. For NS and NSML, mass spectrometry screenings and immunoblots were carried out, but in the case of NF1, mostly low-throughput studies have been performed, targeting only a few proteins [85, 93]. To overcome this lack of information, we aimed to identify potential candidates for phosphoproteomics studies. BRAF phosphorylation is dysregulated both in NS and NSML. The involvement of BRAF in CFC based on genomics studies [8, 9, 11, 14, 21, 23, 60] converts it into an appealing candidate for phosphoproteomic studies. Likewise, RASA1 phosphorylation may be important in CM-AVM [69–71] and PTPN11 in NS, NFNS [8–12] or JMML [42–44]. Other proteins may be good candidates for phosphoproteomic analysis considering their assignation to several syndromes and their high number of phosphosites including SRCAP, A2ML1, ANKRD11, NF1, SOS1 or SOS2.

Noteworthy, most phosphosites in RASopathy proteins are reported on high-throughput mass spectrometry analysis. Protocols rely on different enrichment methods for phosphoproteins (once trypsinization, S-alkylation and reduction are performed). Nano-LC–MS/MS with reversed phase columns efficiently detects phosphopeptides and the modified amino acid, although occasionally this information is limited. Strategies of isobaric labelling (TMT, iTRAQ, SILAC) offer relative quantification of phosphorylated forms or, at least, qualitative information. For instance, the iTRAQ quantifications on SHP2 mutants [90–92] and immunoprecipitated BRAF phosphomapping [94] for NS and NSML, or SILAC strategies in *NF1*-null cells [89]. On the other hand, low-throughput analyses include immunoblots with antibodies designed towards specific phosphoresidues in purified proteins as documented for RAF-1 [86] or CRMP-2 [93]. Commercially-available kits for western-blotting of phosphorylated proteins as ProQ-Diamond dying have also been used [93], although information of the phosphoresidue must be obtained somehow else. This is the case of the two undefined phosphosites in PZR and SIRPa in our dataset. High-throughput mass spectrometry analysis broadens, with its sensitivity limitations in the pmol range, to the examination of the whole phosphoproteome, whereas immunoblotting is limited to a few chosen proteins. Nonetheless, immunoblot analAysis offer specific and reliable information that may complement the monitorization of hundreds of proteins identified by MS strategies.

## Conclusions

Herein, we provide for further studies with a comprehensive up-to-date analysis of 27 RASopathy genes involved in the RAS-MAPK pathway and their interactome, which comprises at least 432 different proteins including 12 nodes, highlighting AKT, HRAS and PIK3R1 as the top three, and involved in several biological processes directly related with the nervous system including but not limited to axonogenesis, peripheral nervous system development and neuron apoptosis.

Current state-of-the-art allows obtaining rich information based on the hypothesis that phosphoproteome remodeling is a key event in RASopathies. Accordingly, we present an integrative and comprehensive analysis of their interactome with regards to dysregulated phosphoproteins. 370 out of the 432 proteins are annotated as phosphoproteins, from which we identify a set of 37 phosphoproteins dysregulated in NF1 (3 proteins), NS (33 proteins), NSML (19 proteins), NSL (1 protein), NSLH (3 proteins), LS (2 proteins), CS (2 proteins), CFC (3 proteins) and JMML (2 proteins), some of them found in more than one syndrome. For most RASopathies, phosphorylation of downstream effectors was upregulated compared to the control, including but not limited to AKT, MEK1 and ERK1/ERK2. All RASopathies are associated to at least one phosphoprotein, in which the NS leads the number of identified phosphoproteins with 13 proteins, followed by NFNS (5 proteins) and JMML (5 proteins). Although just a few phosphoproteomic assays have been done in the onset of RASopathies, we encourage to outline future strategies to unveil the molecular events underlying the RASopathies using mass spectrometry approaches. This is an exciting field to widen molecular knowledge in rare diseases and even aim for translation into clinics.

## Methods

### Generation of a RASopathy proteins interactome

STRING (https://string-db.org/) is a database of known and predicted protein–protein interactions. The interactions include direct (physical) and indirect (functional) associations; they stem from computational prediction, from knowledge transfer between organisms, and from interactions aggregated from other (primary) databases. In order to analyze the RASopathy proteins using STRING, we first compiled an updated panel of RASopathy-related genes including 27 RASopathy genes: *A2ML1*, *BRAF*, *CBL*, *HRAS*, *KAT6B*, *KRAS*, *LZTR1*, *MAP3K8*, *MEK1*, *MEK2*, *MRAS*, *NF1*, *NRAS*, *PPP1CB*, *PTPN11*, *RAF1*, *RASA1*, *RASA2*, *RIT1*, *RRAS*, *RRAS2*, *SHOC2*, *SOS1*, *SOS2*, *SPRED1*, *SPRY1* and *SYNGAP1*; and 5 extra genes *ANKRD11*, *FGFR3*, *MEF2C*, *SHOX* and *SRCAP*, that were previously proposed as RASopathy proteins as mentioned in the introduction. The UniProt IDs for all the above 32 proteins were submitted to STRING database under the multiple proteins search. The following basic settings were used to generate a portable network graphic: confidence for the meaning of network edges (line thickness indicates the strength of data support); active interaction sources are based on experiments and databases only; and high confidence (0.700) as the minimum required interactome score. Edges represent protein–protein associations. For the generation of the RASopathy proteins interactome (Fig. 2A) in STRING “none/query proteins only” and “none” values were used for the 1st and 2nd shell, respectively. These associations are meant to be specific and meaningful (i.e. proteins jointly contribute to a shared function), although this does not necessarily mean they are physically binding each other.

### Generation of a RASopathy proteins and neighbors interactome

An interactome was generated including the above 32 RASopathy proteins and their neighbors. We first downloaded all protein–protein associations for each individual RASopathy protein from STRING (using export your current network protein annotations link) and complemented with those annotated in UniProt database (under the Interaction section, which provides information on interaction(s) with other proteins or protein complexes). Data compilation yielded a total of 765 proteins (Additional file 1: Tab1), including 511 unique proteins based on UniProt IDs (Additional file 1: Tab2). Functional annotation of the 511 unique proteins was carried out using DAVID (https://david.ncifcrf.gov/).

### Analysis of the main hub proteins of the interactome network

In order to further analyze relationships within the whole interactome derived from the 32 RASopathy proteins, we used STRING to build a network of the interactions with the above list of 511 unique proteins (Additional file 1). The resulting network has a total of 8191 edges representing protein–protein associations. Then, in order to find highly connected nodes within the network, the number of edges for every single node was calculated. We focused our analysis on selected nodes that had a number of interactions greater than 4 times the average of interactions found in the network (mean = 18.4 interactions).

### Dataset compilation of dysregulated phosphosites in RASopathies

We scrutinized all previous phosphoproteomics studies carried out in RASopathies using PubMed with the following keywords: “phosphoproteomics”, “phosphoproteome”, “phosphorylation”, “RASopathy”, “Neurofibromatosis”, “Noonan”,”Leopard”, “cardiofaciocutaneous”, “Legius”, “Costello”, “autism”, “capillary malformation-arteriovenous malformation syndrome”, “juvenile myelomonocytic leukemia”, “NF1”, “PTPN11”, “RAF1”, “MEK1”, “BRAF”, “KRAS”, “MEK2”, “SPRED1”, HRAS “A2ML1”, “CBL”, “MAP3K8”, “KAT6B”, “RRAS”, “SPRY1”, “SHOC2”, “PPP1CB”, “SYNGAP1”, “RASA1”. Protein phosphorylation level changes associated to RASopathies were compiled from nine phosphoproteomic and twenty-seven immunoblotting studies (Additional file 4) [15, 16, 19, 21, 85–87, 89–94, 120–138]. The sample size, animal model, type of data (quantitative/qualitative) and statistical validation is also included in Additional file 4. These phosphosites were compiled (Additional file 4) and belong to different organisms including human, monkey, rat, mouse and zebrafish. Phosphoproteins in the original organism were correlated to the orthologous human counterpart. UniProt retrieve/ID mapping tool [139] was used for mouse, rat and monkey proteins, and ZFIN [140] database was used for zebrafish proteins. The equivalent phosphosites in human were mapped by pairwise comparison of the phosphosite orthologous sequences, using the Phosphosite database [141]. Each identified dysregulated phosphosite was annotated based on the human UniProt protein name and the phosphorylated residues.

### Analysis of dysregulated phosphoproteins present in the RASopathy interactome

Differential phosphorylation levels were analyzed in the RASopathy interactome. UniProt IDs from the interactome (Additional file 1) and from the dataset of dysregulated phosphosites in RASopathies (Additional file 4) were crossed. The identified proteins were selected for further analysis. Phosphorylation levels are normalized to the total abundance of each phosphoprotein, providing harmonization of the data between experimental models and organisms. Fold changes in the RASopathy versus control were used to represent dysregulation in each phosphosite, transformed to base 2 logarithm (*log2[fold change]*). A threshold of 1.5 both in upregulation and downregulation was applied to filter out noise in the data. Qualitative data was processed in parallel assigning up- and down-regulation based on original data. R 3.63 [142] and RStudio 1.2.5042 [143] were used to generate a heatmap representing fold changes of each phosphosite (R package pheatmap [144]). Clustering analysis was done in NS and NSML by selecting manually from quantitative data dysregulated phosphoproteins with available information for only NS syndrome, phosphoproteins equally dysregulated in both syndromes and phosphoproteins dissimilarly dysregulated in both syndromes.

The analysis of dysregulated phosphoproteins present in the RASopathy interactome presented some limitations. Different models, even when they mimic the same syndrome, may show a different response in terms of phosphorylation changes that may be driven in part, by the different experimental conditions. Therefore, experimental validation is recommended for those less studied phosphoproteins. In terms of statistical validation, some original phosphoproteomic studies [89–92] were done for screening purposes. Most immunoblots data was statistically validated including several biological replicates. These aspects are included in Additional file 4.

### Analysis of RASopathy proteins phosphosites

We investigated the presence of phosphosites in the 27 confirmed RASopathy proteins using available information in the Phosphosite [141] database. All phosphosites up to available referenced works demonstrating phosphorylation of amino acids in the human sequence were included, independently of the study on their dysregulation in RASopathies (Additional file 6). Classification of the references into high-throughput and low-throughput studies was annotated, but both equally considered. Total number of phosphosites per protein with correlation to the cognate RASopathy syndrome, were represented in a bubble plot using R 3.63 [142] and RStudio 1.2.5042 [143] (R package ggplot2).

### Gene ontology analysis and biological processes

The 27 RASopathy proteins (Fig. 3B) and the 33 dysregulated phosphoproteins in the RASopathy interactome (Fig. 4B) were annotated for GO terms using PANTHER (http://www.pantherdb.org/) classification system tool. A statistical over-representation test using the human PANTHER GO-Slim Biological Process database was used. All groups were selected attending to its statistical significance (FDR value < 0.01) and according to the most specific subclass on the GO hierarchy term they belong to.

### Limitations of the study for phosphoproteomics data

In its conceptualization, our study compiles information from articles including different models and statistical validations assuming some limitations. MS phosphoproteomics studies were used for screening purposes [89–92], and statistics are limited since no p-value test has been performed. Qualitative data from immunoblots is validated by replication of the experiment and fullfill a p-value threshold of 0.05. Details for each study can be consulted in Additional file 4.Therefore, we can only suggest herein the impact of phosphorylation changes in RASopathies and key phosphoproteins from a point of view of screening and based in the co-occurrence and relevance considering crossed data.

## Supplementary Information

**Additional file 1: Table S1.** The direct interactome of the RASopathy proteins.

**Additional file 2: Table S2.** Overlap of the direct interactome of the RASopathy proteins.

**Additional file 3: Table S3.** Functional annotation clustering of the RASopathy interactome proteins.

**Additional file 4: Table S4.** Dysregulated phosphoproteins in RASopathy animal models.

**Additional file 5: Table S5.** Dysregulated phosphorylation in the RASopathy interactome.

**Additional file 6: Table S6.** Phosphosites of RASopathy proteins.

## Data Availability

The dataset(s) supporting the conclusions of this article are included within the article (and its Additional File(s)).

## Abbreviations

NF1: Neurofibromatosis type I
LC-MS: Liquid chromatography coupled to mass spectrometry
LS: Legius syndrome
NS: Noonan syndrome
NFNS: Neurofibromatosis-Noonan syndrome
NSL: Noonan syndrome-like
NSML: Noonan syndrome with multiple lentigines
NSLSH: Noonan syndrome-like with loose anagen hair
CS: Costello syndrome
CFC: Cardiofaciocutaneous syndrome
CM–AVM: Capillary malformation–arteriovenous malformation syndrome
JMML: Juvenile myelomonocytic leukemia
MPNST: Malignant peripheral nerve sheath tumors
GEFs: Guanine nucleotide exchange factors
GAPs: GTPase-activating proteins
